# An explainable machine learning approach for Alzheimer’s disease classification

**DOI:** 10.1038/s41598-024-51985-w

**Published:** 2024-02-01

**Authors:** Abbas Saad Alatrany, Wasiq Khan, Abir Hussain, Hoshang Kolivand, Dhiya Al-Jumeily

**Affiliations:** 1 School of Computer Science and Mathematics, Liverpool John Moores University, Liverpool, UK; 2 University of Information Technology and Communications, Baghdad, Iraq; 3 Imam Ja’afar Al-Sadiq University, Baghdad, Iraq; 4 NIHR Leicester Biomedical Research Centre, University of Leicester, Leicester, UK; 5 Department of Electrical Engineering, University of Sharjah, Sharjah, United Arab Emirates

**Keywords:** Health care, Mathematics and computing

## Abstract

The early diagnosis of Alzheimer’s disease (AD) presents a significant challenge due to the subtle biomarker changes often overlooked. Machine learning (ML) models offer a promising tool for identifying individuals at risk of AD. However, current research tends to prioritize ML accuracy while neglecting the crucial aspect of model explainability. The diverse nature of AD data and the limited dataset size introduce additional challenges, primarily related to high dimensionality. In this study, we leveraged a dataset obtained from the National Alzheimer’s Coordinating Center, comprising 169,408 records and 1024 features. After applying various steps to reduce the feature space. Notably, support vector machine (SVM) models trained on the selected features exhibited high performance when tested on an external dataset. SVM achieved a high F1 score of 98.9% for binary classification (distinguishing between NC and AD) and 90.7% for multiclass classification. Furthermore, SVM was able to predict AD progression over a 4-year period, with F1 scores reached 88% for binary task and 72.8% for multiclass task. To enhance model explainability, we employed two rule-extraction approaches: class rule mining and stable and interpretable rule set for classification model. These approaches generated human-understandable rules to assist domain experts in comprehending the key factors involved in AD development. We further validated these rules using SHAP and LIME models, underscoring the significance of factors such as MEMORY, JUDGMENT, COMMUN, and ORIENT in determining AD risk. Our experimental outcomes also shed light on the crucial role of the Clinical Dementia Rating tool in predicting AD.

## Introduction

Alzheimer’s disease (AD) is a neurodegenerative disorder that affects memory, thinking, and behaviour over time. It is the most common cause of dementia in older adults^1^ and is characterised by the abnormal amyloid beta protein and tau protein accumulation in the brain. These protein abnormalities cause brain cell death and loss of brain function. AD symptoms typically begin with mild memory loss and difficulty in completing familiar tasks^2^. As the disease progresses, symptoms such as difficulty in communicating, disorientation, mood, behaviour changes, and inability to care for oneself, may become more severe^3^.

The machine learning (ML) techniques have been used in the field of AD to analyse various types of data for the identification of distinguishing patterns associated with the disease. Previously, ML have been utilised for the AD detection and diagnosis to identify individuals at risk of developing AD before the onset of significant symptoms^4–8^. This might be useful for the earlier intervention, potentially slowing disease progression. Similarly, ML has also been used to analyse genetic data^9–12^ which may aid in identifying individuals who are more likely to develop AD, allowing for targeted prevention or early intervention strategies.

While ML models have demonstrated efficiency in a variety of medical applications, their lack of transparency poses challenges for real-world healthcare settings. Consequently, ML techniques in the clinical domain often do not employ sophisticated models, instead opting to use simpler, interpretable statistical models (e.g. linear regression), that may have limitations in achieving high accuracy^13^. Literature contains several works to analyse the complex models and open the black box of decision-making processes^14^ however, very few recent research have focused on the interpretability and explainability of ML models in AD. To become acceptable and trusted by physicians (and clinical experts), these models must be comprehensible and retraceable. Therefore, it is crucial for these ML models to provide interpretation of the specific medical decision-making process or diagnostic task they perform.In this regard, an explainable Artificial Intelligence (AI) model is presented in a research study^15^ using structural analysis of AD. Through the use of a modified version of deep BrainNet, this study classified the subjects with AD, Mild cognitive impairment (MCI), and Normal Controls (NC). Using the model in an ablation analysis, brain regions and their connectivity involved in AD was identified by measuring how regions and edges affect neural network prediction, and visualising the brain regions driving the majority of neural network output.

The authors in^16^ proposed three explainable deep learning architectures to analyse language abilities to detect individuals with AD. Each architecture uses different features such as part-of-speech features, language embedding features, and both via a unified architecture. Two types of model explanations were generated: in an intra-class explanation, the relative importance of each of the various features in a class is captured, but in the inter-class explanation, the relative importance of features between classes. An explainable deep learning model for AD was presented in work^17^. Using 3D GradCAM, an attribution-based method was used to explain the decisions made by the model. It also investigated whether GradCAM could affect the heatmaps produced by various convolutional layers of the network. A recent work presented in^18^ used ML to investigate factors with significant impact on AD occurrence and progression. An XGBoost model was used to discriminate different stages of the disease producing a classification F1-score of 84%. A SHapley Additive exPlanations (SHAP) model is used on top of the trained ML model to produce both local and global explanations. Similar work is presented in work^19^ which uses SHAP model in addition to a Random Forest (RF) classifier to classify: NC, cognitive impairment and dementia using cognitive scores as input.

Danso et al.^20^ used two tree-based algorithms to build ML models on a dataset from the European population to predict the risk of AD, then transfer learning the best model on another dataset from the UK population. Furthermore, they apply SHAP to visualize population-based and individual-level risk factors. A two-layer RF model approach was proposed in study^21^ for diagnosis and progression detection of AD. The first layer acts as a multi-classification to detect AD from NC and MCI. Whereas the second layer acts as a prediction tool to forecast the progression from MCI to AD. Their model is trained and tested using various biological and clinical attributes of 1048 individuals. The SHAP algorithm is used to provide global and individual-based explanations of RF classifiers for each layer.

The above literature shows variety of approaches addressing the explainable ML models for the AD classification. However, few studies’ reliance on a single ML algorithm may raise concerns about the robustness and generalizability of the results. It would be beneficial to compare the performance of other algorithms. While some studies relay on a single type of data, it fails to capture the full complexity of the disease. Therefore, by incorporating multimodal data, ML models can capture a broader range of AD-related information and uncover hidden patterns and biomarker correlations that may not be apparent in a single data type.

Most studies utilise SHAP to explain the ML models. SHAP aims to provide local and global explanations for ML predictions, helping to interpret the factors contributing to the model’s decisions. However, when the AI model itself lacks transparency or interpretability, SHAP may face challenges in producing meaningful and reliable explanations. Additionally, the quality and accuracy of the explanations heavily depend on the features and data used to train the ML model. In cases where the features or data do not adequately represent the complexity of AD or capture relevant biological factors, the explanations provided by SHAP may be limited in their ability to provide comprehensive insights.

Despite the considerable amount of research conducted, its impact on clinical practice is often limited due to several reasons. Firstly, many studies rely exclusively on a single method of analysis, particularly neuroimaging. This narrow focus may overlook valuable information from other modalities. Secondly, the emphasis on improving the accuracy of ML models has overshadowed the importance of its explainability which poses challenges in clinical settings where practitioners may not be familiar with the machine-based complex analysis and decision making. Additionally, ML models often require large amounts of data to achieve accurate predictions, which may pose challenges in real-world applications.To address these limitations, the proposed study presents reliable ML algorithms to classify different cognitive states of a person, with following contributes:

- Leveraging extensive data: Utilization of a big dataset of comprising 169408 observations and 1024 features. This extensive dataset provides a robust foundation for our research.
- Accurate Multiclass Classification: classifications of individuals into multiple AD classes, including NC, MCI and AD with high and balanced performance.
- Long-term cognitive state prediction: Developing a model capable of predicting the cognitive state of an individual four years after their baseline visit. This prognostic capability has significant implications for early intervention and personalised treatment strategies.
- Rule extraction in AD classification: This the first time in literature, SIRUS or CAR algorithms have been applied to AD classification. Through these models, we extract human-understandable rules that elucidate the inter-relationships between the most significant factors contributing to development of AD.

## Results

Detailed results as retrieved from the various experiments (section “Experiment design”). For each experiment, results are shown from multiple classifiers that include RF, k-nearest neighbour (KNN), Naïve Bayes (NB) and support vector machine (SVM). For each classifier, detailed metrics are retrieved to compare the classifiers’ performances in corresponding experiments that are described as follows.

### Results for EXP1

Table 1 presents the results of EXP1 which utilises original features (i.e., all features without feature reduction). It can be noticed from Table 1(a) that the highest accuracy of 97.8% was achieved by the RF algorithm for the classification of NC against AD cases when evaluated over unseen data samples. Furthermore, the RF model indicated robust performance for other metrics such as precision, recall, and F1 (97.2%, 98.1% and 97.6%, respectively), indicating its ability to provide stable and balanced classification with fewer false classifications among both classes. These outcomes suggest the RF model as an effective tool for the classification of NC and AD cases, with a high degree of accuracy and reliability.

Table 1(b) presents the results of the performance of classifiers in classifying NC and MCI cases. Among the different classifiers, RF achieved the highest accuracy of 88.6%. On the other hand, the KNN classifier indicated poor performance, with an imbalanced precision and recall of 81.2% and 48% respectively. This demonstrates that KNN is not an ideal model for classifying NC and MCI cases.

Table 1(c) shows the results for classification between MCI and AD cases, indicating RF as outperforming classifier. This is in agreement with the results of Table 1(a, b), which also shows that the RF is the best performing classifier. On the other hand, the NB classifier shows comparatively poor performance with an accuracy of 82.4%. Furthermore, the NB is biased in terms of precision and recall of 92.5% and 76.4%, respectively.

Table 1(d) shows the final results of EXP1, where the classifiers are trained and tested over a multi-classification problem to classify three classes including NC, MCI, and AD. We used one-vs-one strategy^22^ where the multi-class classification task is broken up into a series of binary classification problems and was chosen over the alternative strategies as it provides improved performance. It can be noticed that the RF algorithm again outperformed (with 85.2% accuracy) followed by the SVM (85.1%) and KNN with least performance (with accuracy of 75.5%). This is likely due to KNN not being able to capture the complexity of the data of three classes. Additionally, the performance of the RF model was consistent across all metrics, making it a reliable and robust choice for any classification task.

Overall, it can be observed that the classifiers achieved better results when classifying NC vs AD (Table 1(a)) compared to NC vs MCI (Table 1(b)) and MCI vs AD (Table 1(c)). This is not surprising, given that NC cases are closer in terms of characteristics to MCI, and MCI and AD are also similar. However, the classification between NC and AD is easier to carry out due to the significant differences between the two. For example, the cognitive decline in AD is much more pronounced than in NC, making it easier for the classifiers to differentiate between the two.

**Table 1:** Results of EXP1.

ML Model	Accuracy%	Precision%	Recall%	F1 score%	Mean%	SD	P-value
(a) Results of EXP1 : NC vs AD							
RF	97.8	97.2	98.1	97.6	97.8	0.002	
KNN	94.8	97.8	90.8	94.1	94.2	0.003	P<0.001
NB	96.2	93.8	98.3	96	96.1	0.002	P<0.001
SVM	97.6	97.6	97.2	97.4	97.6	0.003	P = 0.292
(b) Results of EXP1 : NC vs MCI							
RF	88.6	81.9	88.6	85.1	85.9	0.003	
KNN	76.8	81.2	48	60.3	59.5	0.006	P<0.001
NB	82.4	76.8	74.7	75.8	76.1	0.005	P<0.001
SVM	88.1	82.1	86.7	84.3	85.3	0.003	P = 0.003
(c) Results of EXP1 : MCI vs AD							
RF	87.3	90.2	88.1	89.1	90.5	0.002	
KNN	83.1	89.6	81	85	86.7	0.002	P<0.001
NB	82.4	92.5	76.4	83.7	85.6	0.004	P<0.001
SVM	87.6	90.4	88.5	89.4	90.3	0.003	P = 0.49
(d) Results of EXP1 : NC vs MCI vs AD							
RF	85.2	85.6	85.2	85.4	86.3	0.002	
KNN	75.5	74.1	75.5	73.4	73.4	0.005	P<0.001
NB	77.9	78.7	77.9	78	79.1	0.001	P<0.001
SVM	85.1	85.3	85.1	85.2	86	0.004	P = 0.20

Performance of ML models in classifying: (a) NC vs AD, (b) NC vs MCI, (c) MCI vs AD and (d) NC vs MCI vs AD. For each task, we employed five-fold cross-validation on the training data. Four folds were used for training, and the remaining fold was used for testing, resulting in five replicas. Statistics were derived using the F1 score. We conducted a performance comparison between RF and the other models to determine the presence of statistically significant differences. P-values were calculated using a two-sided t-test, and the means and standard deviations are listed in the table. Subsequently, we internally evaluated the model by training it on the entire training dataset and testing it on a hold-out test dataset, with the results reported in the table.

### Results for EXP2

As described in the Experiment section , EXP2 evaluates the performance of four ML models in classifying three groups of subjects: NC, MCI, and AD while using the reduced set of features (Supplementary Figs. 9, 10, 11 and 12) as produced by Algorithm1. The results of the classification are presented in Table 2.

Table 2(a) presents the classification results for NC vs AD, where all models achieved high performance with accuracy above 96%. The RF model performed the best with an accuracy of 97.5%, followed by SVM with an accuracy of 97. 3%. In terms of precision and recall, all models performed almost similar with scores above 94%. Overall, the results suggest that the ML models are capable of accurately distinguishing between NC and AD subjects utilising the reduced features. Table 2(b) shows the classification results for NC vs MCI, where the RF and SVM models achieved same accuracy rates of 88.1%, while the KNN and NB models should a slightly reduced accuracy rate. RF model achieved highest recall score but showed a marginally reduced precision comparing to other classifiers.

Table 2(c) presents the classification results for MCI vs AD, where all models achieved accuracy rates above 82%. The NB model achieved the highest precision score, while the RF model achieved the highest recall score. Table 2(d) shows the classification results for multi-class classification of NC vs MCI vs AD, where all models achieved accuracy rates above 78%. The SVM model performed the best, achieving performance rates above 84%. The NB model achieved the lowest accuracy rate among the four models. The SVM model also achieved high precision and recall scores across all classes.

In summary, the ML models indicate reliable performance in classifying NC, MCI, and AD subjects. The RF and SVM models consistently achieved high accuracy rates and precision and recall scores across all classification tasks, suggesting that they are effective models for the task of AD classification.

**Table 2:** Results of EXP2.

ML Model	Accuracy%	Precision%	Recall%	F1 score%	Mean%	SD	P-value
(a) Results of EXP2 : NC vs AD							
RF	97.5	97	97.6	97.3	97.5	0.002	
KNN	96.4	97.1	95.2	96.1	96.6	0.002	P<0.001
NB	96.1	94.2	97.5	95.8	96.4	0.001	P<0.001
SVM	97.3	97.1	97.1	97.1	97.5	0.002	P = 0.846
(b) Results of EXP2 : NC vs MCI							
RF	88.1	81.3	87.7	84.4	89	0.003	
KNN	87.5	81.6	85.4	83.5	88.4	0.006	P = 0.158
NB	82.9	83.6	66.5	74.1	83	0.004	P<0.001
SVM	88.1	82.1	86.7	84.3	89	0.002	P = 0.821
(c) Results of EXP2 : MCI vs AD							
RF	86	88.7	87.4	88.1	87	0.003	
KNN	84.4	89.6	83.3	86.3	85.4	0.005	P = 0.001
NB	82.4	93.4	75.7	83.6	84.3	0.006	P<0.001
SVM	86.6	90.4	86.6	88.5	87.6	0.002	P = 0.028
(d) Results of EXP2 : NC vs MCI vs AD							
RF	82.6	82.9	82.6	82.7	85.3	0.002	
KNN	82.5	83	82.5	82.7	82.7	0.004	P<0.001
NB	78.2	78.6	78.2	78.1	79.2	0.002	P<0.001
SVM	84.7	85.2	84.7	84.9	85.7	0.004	P = 0.185

Performance of ML Models using reduced feature sets in Classifying: (a) NC vs AD, (b) NC vs MCI, (c) MCI vs AD and (d) NC vs MCI vs AD. For each task, we employed fivefold cross-validation on the training data. Four folds were used for training, and the remaining fold was used for testing, resulting in five replicas. Statistics were derived using the F1 score. We conducted a performance comparison between RF and the other models to determine the presence of statistically significant differences. P-values were calculated using a two-sided t-test, and the means and standard deviations are listed in the table. Subsequently, we internally evaluated the model by training it on the entire training dataset and testing it on a hold-out test dataset, with the results reported in the table.

### Results for EXP3

In Exp 3, we employed ML classifiers to predict an individual’s cognitive state four years after their initial visit. To assess the accuracy of our classifiers, we conducted a series of experiments, the outcomes of which are detailed in Table 3. In the binary classification task of distinguishing between NC vs AD, all models achieved notably high accuracy with RF excelled with the highest accuracy of 96.4 while NB exhibited a slightly reduced accuracy of 95.1%.

In the binary classification task of NC vs MCI, all models achieved accuracy rate exceeding 71%, with RF achieving the best accuracy and F1 score, measuring 78.1% and 75.7%, respectively. Furthermore, all ML models demonstrated imbalanced performance in terms of precision and recall. For instance, NB reached precision and recall scores of 90.4% and 48.5%, respectively. Conversely, RF showed the least biased performance, with precision and recall scores of 85.9% and 67.6% respectively.

In the binary classification of MCI and AD, RF achieved the highest accuracy of 76.7% along with the highest, recall and F1 score, measuring 82% and 78%, respectively. However, NB achieved the highest precision of 90.1% but indicated a comparatively lower recall score of 64%.

In the multi-class classification task encompassing NC, MCI and AD, RF achieved the highest F1 score of 72.6% and maintained stable performance in terms of precision and recall of 72.5% and 73%, respectively. Conversely, NB indicated least F1 score of 67%.

The results presented in Table 3 underscore the classifiers’ ability to more accurately predict NC and AD classes compared to the MCI class. This outcome aligns with expectations, given that distinguishing between NC and AD classes is typically more straightforward, whereas MCI falls in an intermediate category, presenting a greater challenge.

**Table 3:** Results of EXP3.

ML Model	Accuracy%	Precision%	Recall%	F1 score%	Mean%	SD	P-value
(a) Results of EXP3: NC vs AD							
RF	96.4	97.5	95.1	96.3	95.2	0.009	
KNN	95.8	97.8	93.4	95.5	90.6	0.017	P = 0.001
NB	95.1	94.8	95.1	94.9	94.1	0.011	P = 0.184
SVM	96.1	97.5	94.4	95.9	92.9	0.014	P = 0.029
(b) Results of EXP3: NC vs MCI							
RF	78.1	85.9	67.6	75.7	53.8	0.043	
KNN	72.9	83.1	58	68.3	42.3	0.022	P = 0.001
NB	71.4	90.4	48.5	63.1	53.9	0.029	P = 0.892
SVM	75.9	85.1	63.2	72.5	54.9	0.036	P = 0.729
(c) Results of EXP3: MCI vs AD							
RF	76.7	74.5	82	78	89.5	0.009	
KNN	74.2	74.2	75	74.6	87.2	0.008	P = 0.007
NB	78.2	90.1	64	74.8	79	0.021	P<0.001
SVM	76.2	74.7	80	77.2	90.3	0.008	P = 0.249
(d) Results of EXP3: NC vs MCI vs AD							
RF	73	72.5	73	72.6	76.5	0.005	
KNN	69.8	69.2	69.8	69.4	72.2	0.006	P<0.001
NB	67.8	68.1	67.8	67	73.1	0.013	P = 0.001
SVM	71.6	71.2	71.6	71.4	73.3	0.012	P = 0.002

Performance of ML Models using reduced feature sets in predicting: (a) NC vs AD, (b) NC vs MCI, (c) MCI vs AD and (d) NC vs MCI vs AD. For each task, we employed fivefold cross-validation on the training data. Four folds were used for training, and the remaining fold was used for testing, resulting in five replicas. Statistics were derived using the F1 score. We conducted a performance comparison between RF and the other models to determine the presence of statistically significant differences. P-values were calculated using a two-sided t-test, and the means and standard deviations are listed in the table. Subsequently, we internally evaluated the model by training it on the entire training dataset and testing it on a hold-out test dataset, with the results reported in the table.

### Results for EXP4: external validation

To assess the generalizability of the classifiers, we conducted an external validation using the ADNI Dataset, testing the two top-performing classifiers, RF and SVM across a range of tasks. These tasks encompassed the classification of cognitive states at the baseline visit and the prediction of cognitive states 4 years later, including CN vs AD, CN vs MCI, MCI vs AD, and CN vs MCI vs AD.

The outcomes of Experiment 4, detailed in Table 4, offer insights into the performance of the models. Notably, the classifiers trained for the NC vs AD classification on the NACC dataset exhibited impressive performance when applied to the ADNI dataset. SVM achieved a remarkable 99% accuracy, indicating its superiority, while RF achieved an accuracy of 98.3% (Table 4(a)). However, RF displayed a degree of bias towards precision. In a similar vein, when the models trained for the CN vs AD prediction task on the NACC dataset were tested on ADNI data, both SVM and RF showed higher F1 scores, yet both models demonstrated a degree of bias in terms of precision and recall (Table 4(b)).

SVM proved effective and demonstrated balanced performance in both the classification and prediction of the CN vs MCI subset, as evidenced in Table 4(c, d), respectively. Notably, SVM exhibited a strong performance in classifying MCI vs AD in the ADNI data, achieving an F1 score of 81% (Table 4(e)). However, it showed a drop in performance when tasked with prediction, resulting in an F1 score of 56% (Table 4(f))

Finally, SVM demonstrated balanced and high F1 scores, surpassing 90%, for the classification of CN vs MCI vs AD (Table 4(g)) and maintained a commendable performance in the prediction task, achieving an F1 score of 72.8 (Table 4(h)). These results underscore the versatility and robustness of SVM across various classification and prediction tasks.

**Table 4:** Results of EXP4: performance of RF and SVM in classification and prediction tasks using external ADNI dataset.

ML model	Accuracy%	Precision%	Recall%	F1 score%
(a) Results of EXP4: NC vs AD classification				
RF	98.3	100	80	88
SVM	99	994	984	98.9
(b) Results of EXP4: NC vs AD prediction				
RF	97.8	92.3	80	85.7
SVM	98.3	100	80	88
c) Results of EXP4: NC vs MCI classification				
RF	98.6	99.6	98.4	99
SVM	99.6	99.6	99.8	99.7
(d) Results of EXP4: NC vs MCI prediction				
RF	90.2	86.8	91.3	89
SVM	92.4	91.3	91.3	91.3
e) Results of EXP4: MCI vs AD classification				
RF	87.1	71.6	86.5	78.4
SVM	88.9	75.4	87.5	81
(f) Results of EXP4: MCI vs AD prediction				
RF	66.1	73	51.5	60.4
SVM	64.9	75.7	44.4	56
(g) Results of EXP3: NC vs MCI vs AD classification				
RF	89	90	89	89.2
SVM	90.5	91.4	90.5	90.7
(h) Results of EXP4: NC vs MCI vs AD prediction				
RF	72.9	73.9	72.9	72.6
SVM	73.6	75.2	73.6	72.8

### Results for EXP5: ML explanations and human understandable rules extraction

**Figure 1:**
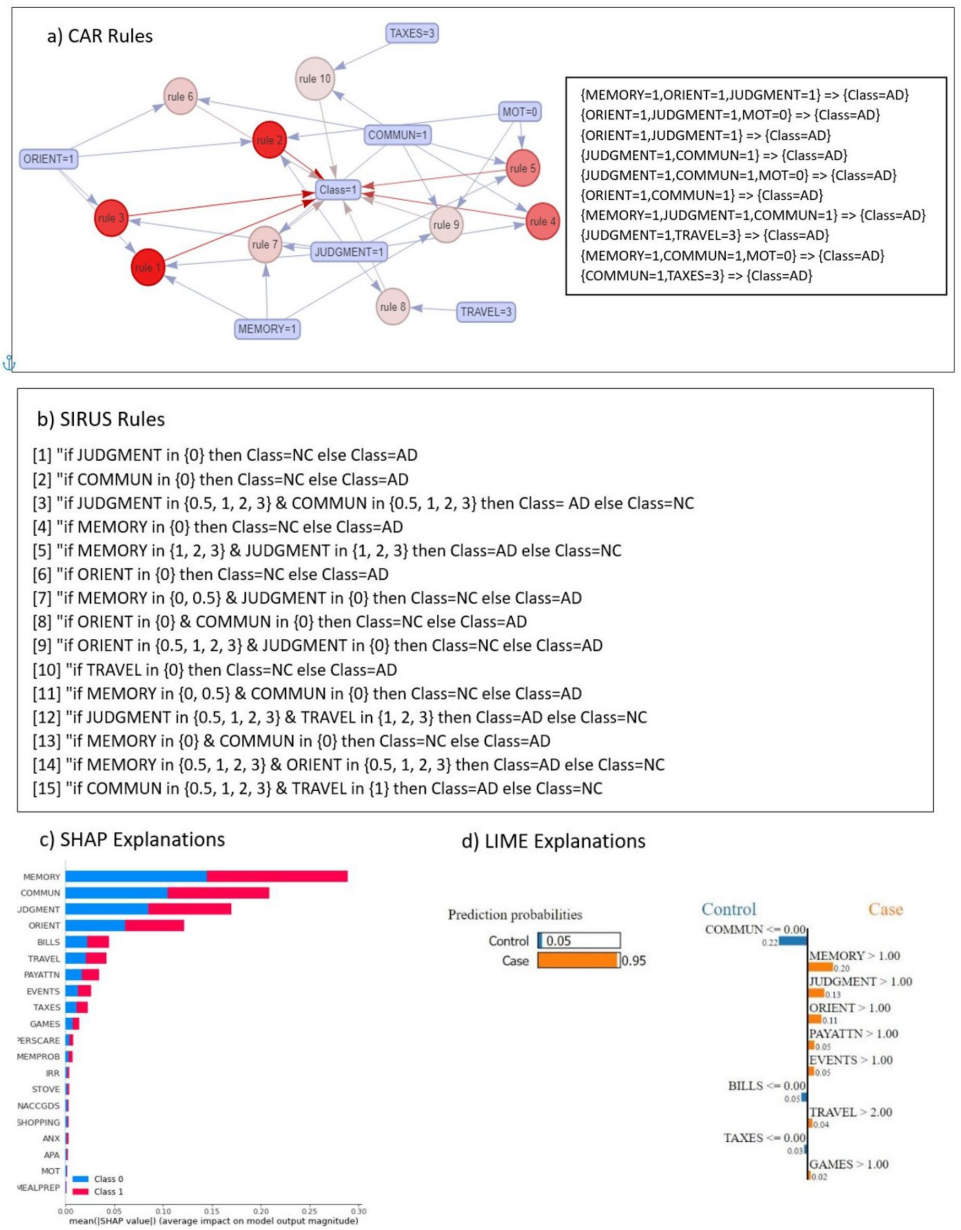
Explanations and rules extraction for NC vs AD subset: (**a**) Visualisation of representative associations and corresponding written rules between multiple factors and AD in NC vs AD, (**b**) list of rules output by SIRUS model, (**c**) explanation provided by SHAP model, (**d**) explanation provided by LIME model for a single instance of the test set.

In our pursuit of understanding the intricate patterns with the data and comprehending the behaviour of ML models in classifying AD, Class Association Rules (CAR) algorithm is used in EXP4. Figure 1a illustrates ten representative rules extracted by CAR that are highly associated with AD. The intensity of the red colour of the circles indicates the strength of the rule, evaluated using the lift measure.

Upon analysis of the output rules, it becomes evident that AD is associated with a wide range of factors, including mild impairments in memory (MEMORY = 1), orientation (ORIENT = 1), judgment and problem-solving (JUDGMENT = 1) and impairments in community affairs (COMMUN = 1). To elucidate the values of the variables (such as MEMORY, JUDGMENT) are encoded as follows: 0 for no impairment, 0.5 for questionable impairment, 1 for mild impairment, 2 for moderate impairment, and 3 for severe impairment. The rules shed light on the combinations of these variables with the severity levels of TRAVEL and TAXES, all of which bear a significant connection to AD.

Stable and Interpretable Rule Set for classification (SIRUS) algorithm was utilised to extract human readable rules and to compare with the rules extracted from CAR. Figure 1b shows the rules extracted from the NC vs AD subset. the first rule indicates that if the value of the variable ‘JUDGMENT’ is ‘0’ the classification is likely ‘NC’ Conversely, if the ‘JUDGMENT’ value is not ‘0’ the likelihood of ‘AD’ classification increases significantly. Essentially, the ‘0’ value for the ‘JUDGMENT’ feature serves as a robust indicator of an individual’s AD status. Similarly, another rule indicates that if the value of the ‘COMMUN’ variable is ‘0’ the individual is most likely classified as ‘NC’ while other values suggest ‘AD’ The rules derived from SIRUS also unveil the co-occurrence of higher values in the TRAVEL, ORIENT, and MEMORY variables, which are associated with an elevated risk of AD.

To validate the rules generated by both models and ascertain the informativeness of these variables in the context of ML AD classification, our research venture extended to encompass the application of two model-agnostic explanation methods: SHAP and Local Interpretable Model-Agnostic Explanations (LIME). As visually depicted in Fig. 1c, d, the variables that SHAP identifies as most informative include MEMORY, COMMUN, JUDGMENT, ORIENT, and BILLS. Concurrently, the insights offered by LIME emphasize the pivotal role of variables such as COMMUN, MEMORY, JUDGMENT, and ORIENT. Table 5 presents the informative features selected by each model, along with the common features chosen by all models. Furthermore, Table 6 demonstrates the performance of SVM when trained and tested using the common features extracted from Table 5. The results of this classifier closely align with the findings of EXP1 and EXP2, underscoring the significance of these features in influencing the model’s performance.

**Table 5:** Features selected from explanations by models for NC vs AD data subset.

Data	Accuracy%	Precision%	Recall%	F1 score%
Feature selected by CAR	Features selected by SIRUS	Features selected by SHAP	Features selected by LIME	Common features selected by all models
MEMORY	JUDGMENT	MEMORY	COMMUN	MEMORY
ORIENT	COMMUN	COMMUN	MEMORY	COMMUN
JUDGMENT	MEMORY	JUDGMENT	JUDGMENT	JUDGMENT
MOT	ORIENT	ORIENT	ORIENT	ORIENT
COMMUN	TRAVEL	BILLS	PAYATTN	
TRAVEL		TRAVEL	EVENTS	
TAXES

**Table 6:** Performance of SVM trained and tested using common features selected by explanation models (from Table 5).

Data	Accuracy %	Precision %	Recall %	F1 score %
NC vs AD	97.2	97.7	96.3	97
NC vs MCI	88.8	79.7	87.2	83.3
MCI vs AD	86	90.4	86.3	88.3
NC vs MCI vs AD	83.5	84.2	83.5	83.8

**Figure 2:**
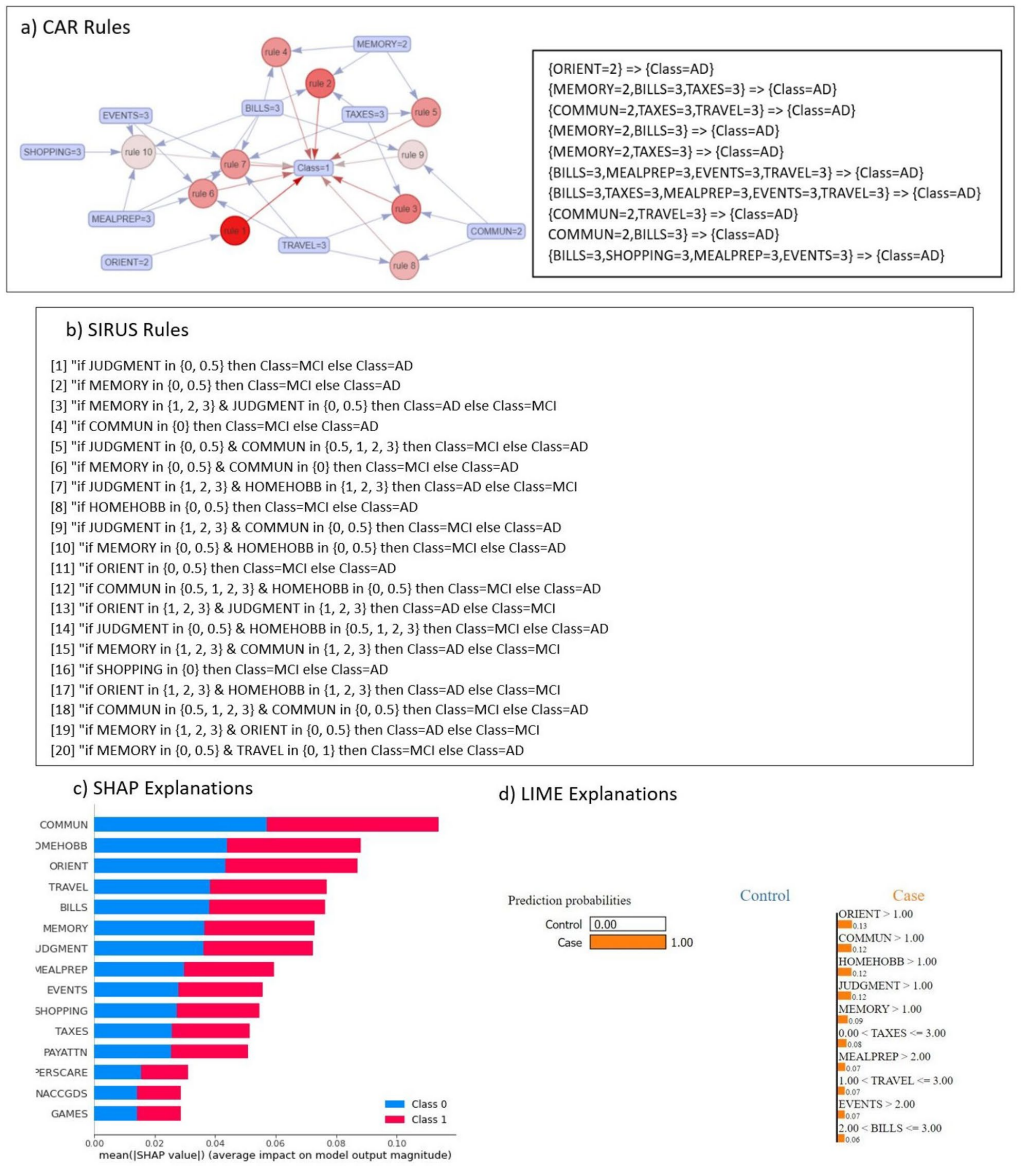
Explanations and rules extraction for MCI vs AD subset: (**a**) Visualisation of representative associations and corresponding written rules between multiple factors and AD in MCI vs AD, (**b**) list of rules output by SIRUS model, (**c**) explanation provided by SHAP model, (**d**) explanation provided by LIME model for a single instance of the test set.

In a similar vein, the patterns discerned from the MCI vs AD data subset are systematically extracted using the CAR algorithm, as depicted in Fig. 2a. This visualization encapsulates ten rules of significance in the context of AD. These rules were selected from a comprehensive number of variables based on their discernible influence on AD. Five pivotal variables-ORIENT, MEMORY, COMMUN, BILLS, and TAXES-emerge as the most robust influencers in the realm of AD. The amalgamation of these variables with elevated values strongly correlates with AD, a consistent pattern observed across both the NC vs AD data subset, as presented in Fig. 1.

Furthermore, the SIRUS algorithm was utilised to extract rules from the MCI vs AD data subset. As elucidated in Fig. 2b, the extracted rules unveil that when the feature ‘JUDGMENT’ assumes a value of either 0 or 0.5, the likelihood of classification as MCI predominates. Conversely, when ‘JUDGMENT’ adopts any other value, the individual’s classification tends toward AD. Similarly, the second rule articulates that when the variable ‘MEMORY’ manifests values of 0 or 0.5, the probability of MCI classification is accentuated. Intriguingly, a high value associated with ‘MEMORY,’ signifying moderate or severe memory impairment, distinctly inclines the individual towards an AD diagnosis. These rules cogently imply that combinations of variables with high values generally align with an AD classification, resonating with the outcomes of the CAR algorithm.

Figure 2c, d offer insights into the explanations provided by SHAP and LIME, respectively. Both models consistently underscore the pivotal roles of COMMUN, ORIENT, and JUDGMENT as informative variables significantly influencing the AD classification, which is in line with both CAR and SIRUS.

## Discussion

The experimental results for Exp1-Exp4 demonstrates the capabilities of ML models in the classification of AD patients from those with NC or MCI. Out of the four models utilised, RF, SVM, NB and KNN, RF and SVM models consistently achieved the highest accuracy, precision and recall scores across all tasks. These models effectively discriminated between NC and AD subjects, as well as between NC and MCI, MCI and AD subjects. While NB and KNN models also demonstrated considerable accuracy, they generally scored lower than RF and SVM.

This indicates the substantial potential of RF and SVM models for accurate AD diagnosis. It is noteworthy that RF has previously shown high accuracy in the analysis of multi-modal data to predict the conversion of MCI to AD^23^. Additionally, in healthcare domain, RF showed better classification of risk assessment of coronary heart disease than other classifiers^24^. These observations can be attributed to the capacity of RF and SVM models to efficiently process large datasets^25^, making them well-suited for large-scale medical diagnoses. Furthermore, these models excel in data generalization, rendering them more adept at handling the intricacies of medical diagnosis. Consequently, RF and SVM models are better positioned to provide AD diagnosis when compared to NB and KNN classifiers.

ML has increasingly been employed in research to predict the progressions of AD stages. For example, work presented in study^26^ developed a hybrid ML framework for the analysis of longitudinal data to predict the prognosis of dementia in patients with MCI. While their model achieved high accuracy of 87.5% using RF, it displayed instability across various performance measures. Notably, the model exhibited a stronger bias towards sensitivity (92.9%) at the expense of specificity, which was only 58.3%. Another study^27^ identified and utilised 15 clinical variables predicting MCI converters reporting 71%, 67.7% and 71.7% for accuracy, sensitivity and specificity, respectively. In contrast, our ML classifiers demonstrate not only high accuracy, precision and recall scores when applied to classification tasks but also achieved robust outcomes when trained and tested as a predictive tool to estimate the cognitive state of a person four years in the future. Specifically, the classifiers excels at identifying subtle changes in cognitive development over time, thus making it a valuable asset in predicting potential changes in cognitive health. Moreover, our approach is found to be reliable and robust, with a high degree of consistency in its predictions over multiple trials (i.e NC vs AD; MCI vs AD; and NC vs MCI vs AD). This means that it can be used to reliably forecast a person’s cognitive state in the future.

The results section provides convincing evidence of the efficacy of SVM in both classification and prediction tasks. SVM performed well, not only when tested on the NACC testing hold subset but also when evaluated on an external ADNI dataset for various tasks (Table 4). This was achieved through the feature selection method (Algorithm 1), which significantly reduced the number of features from 64 to only 21 features. Despite the substantial reduction in feature space, the results demonstrate that the selected features are highly effective in differentiating AD cases.

It is important to note that the objective of this research is not only to obtain better AD classification but also gaining insight into the influential factors that are important for the classifiers’ decision making. To this end, this study conducted a series of experiments to identify the most important features and to understand the underlying relationships that exist between them.

In pursuit of these objectives, we employed two rule extraction methods, CARs and SIRUS, to extract human-readable rules associated with AD. CARs, for instance, utilised seven of the 21 features (refer to Supplementary Fig. 9). While SIRUS used five features to establish its list of most dominant rules. Intriguingly, both algorithms identified common features as shown in Table 5 this overlap strongly suggests that the rules generated by these algorithms exhibit a significant degree of similarity, enhancing the confidence in extracted rules accuracy and reliability. The utilization of two distinct rule extraction methods, with the majority of the rules aligning, underscores the precision and trustworthiness of extracted rules.

Furthermore, the features identified as important by CAR and SUIRS underwent additional validation through SHAP and LIME models, which were utilized to elucidate the decisions made by the top-performing classifier. Notably, both SHAP and LIME consistently identified crucial features that aligned with the rules extracted by CAR and SUIRS (Table 5). This alignment in feature selection across diverse models significantly strengthens the overall robustness and reliability of our findings.

It can be noticed that the CAR is more precise than SIRUS in terms of generating the rules. For instance, the first rule extracted by CAR from the NC vs AD dataset (Fig. 1) specifies that if an individual has the variables MEMERY, JUDGMENT and ORIENT with the value of 1, then it is a case of AD. In contrast, SIRUS, tends to provide generalised predictions. For example, the first rule generated by SIRUS suggest that if the variable JUDGMENT assume the value of 0, then it’s more likely the individual to belong to the class NC. However, if the value of JUDGMENT is not 0 (i.e. 0.5, 1, 2 or 3) then individual is likely to belong to the class AD. This shows that SIRUS can make broader observations and predictions than CAR, which tends to be more specific in its rules.

The findings highlight the collective significance of the features MEMORY, JUDGMENT, ORIENT, and COMMUN are collectively significant in assessing the risk of developing AD as indicated by all models. These combined features play a crucial role in predicting the likelihood of an individual being diagnosed with AD. Literature supports the Clinical Dementia Rating (CDR) as a valuable tool for detecting MCI and AD^28,29^. Research conducted by^30^ underscores the significance of considering functional information, namely JUDGMENT, COMMUN, and HOMEHOBB, as assessed by the CDR, when evaluating individuals with MCI. The intact group included individuals with a rating of 0 in all three categories or a rating of 0.5 in one of the three categories. The impaired group comprised individuals with a rating of 0.5 in two or more of the three IADL categories or a rating of 1 in any one of the categories. The results of the experiments have been instrumental in providing key insights into the efficacy of CDR in the prediction of AD.

## Limitations and future work

In our study, several limitations warrant consideration. Firstly, we conducted feature correlation analysis on discretized continuous values, a process that might result in information loss and potentially affect the precision of correlation assessments. Furthermore, our evaluation hinges on a clinically derived diagnosis label, which might not encompass all the variables considered by our model, thereby leading to an incomplete assessment of the model’s performance.

Our future research will encompass longitudinal analysis, enabling us to delve into how CDR scores evolve over time and explore the potential for predicting future CDR scores. This longitudinal approach holds the promise of providing valuable insights into early AD detection and continuous monitoring. Additionally, our plans involve extending our methodology to broader studies, with a particular focus on forecasting early disease stages, including the transition from non-demented to demented stages. Furthermore, we aim to investigate the utilization of ensemble models that amalgamate multiple explainable algorithms, thereby enhancing the robustness and reliability of our model explanations.

## Methods and materials

**Figure 3:**
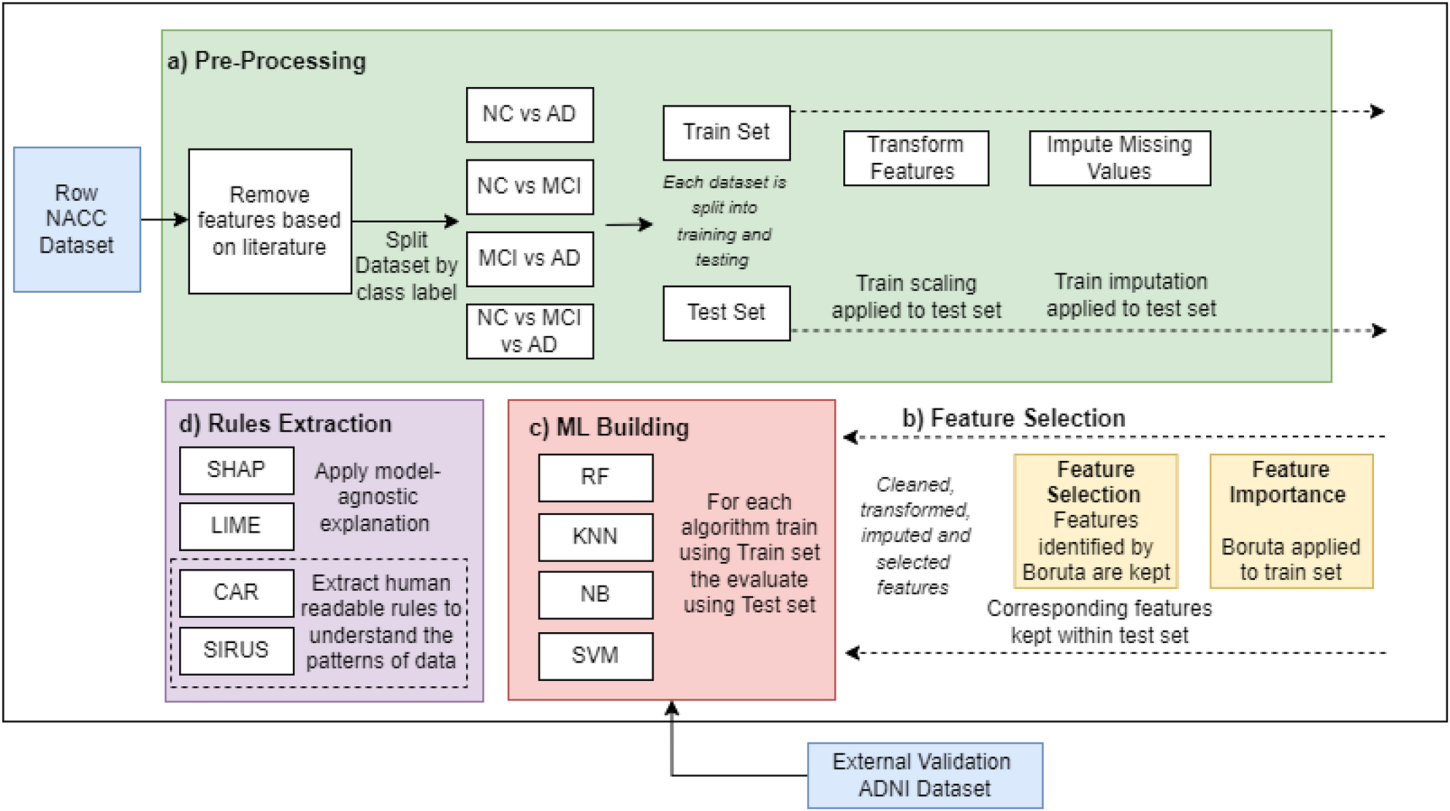
Workflow overview of the proposed methodology. The process begins with data acquisition from NACC and proceeds through several key stages: (**a**) data preprocessing, including the selection of relevant features inspired by existing literature, partition of the dataset based on class labels, division into training and testing subsets, and data transformation and cleansing using the training set as a reference. (**b**) Feature importance is evaluated using the Boruta algorithm, and only the identified features are retained for subsequent analysis. (**c**) Construction of four widely recognized ML classifiers to address various tasks related to the classification of cognitive states. External validation of these models is performed using additional data from the ADNI. (**d**) The final step involves the extraction of human-readable rules from the trained machine learning models, facilitating the interpretation of factors associated with AD.

The proposed research introduces the identification of potential features which are highly associated with the AD progression while utilising a composite of feature selection and ML algorithms. The study further investigates the explainable ML models to extract human understandable insights of complex pattern analysis and machine-based decisions, identifying the potential risk factors for AD. Figure 3 demonstrates the overall proposed methodology. The dataset in this study is requested from National Alzheimer’s Coordinating Center (NACC), which is pre-processed to remove outliers, missing values, and transformation to appropriate form. Dimensionality reduction is then performed using correlation analysis and Boruta algorithm, followed by the implementation of ML models to classify and predict AD. We then use a composite of data analytics approaches to explain the ML model and identify the most significant features. In addition to reliable classification accuracy, this research reveals new insights into the risk factors associated with AD. Furthermore, it aims to explain the ML model by deriving human-understandable rules. These findings could ultimately contribute to enhanced treatments and improved patient care.

### Dataset

#### NACC

The dataset for this study is obtained from NACC and permission for data use has been granted based on the research’s goals set in this study. The original NACC data set is acquired from 45,923 participants comprising 1023 variables. Because NACC contains samples from baseline visit along with follow up visits of participants, the total instances are 169,408 as of August 2022. In general, the dataset attributes are derived from different factors including demographics, diagnoses, neuropsychological tests, and clinical assessments. Cognitive functions were evaluated employing a standardized neuropsychological battery^31^. The mini-mental state examination (MMSE) was utilized to measure overall cognitive functioning of subjects.

The data samples were labelled by medical experts according to the description of the data set. Participants are classified using the NACC variable NACCUDSD, which indicates the level of cognitive impairment ranging from normal cognition to MCI and AD.

Table 7 presents a summary of demographic information. The average age of patients at their initial visit to the AD Center was 72 years. A significant proportion of the patients were female. Notably, higher mean BMI, heart rate, and blood pressure were observed to be positively associated with cognitive state levels at the time of their initial presentation. More information regarding the NACC data set can be found in^32^.

**Table 7:** Demographic information for NACC participants.

	NC	MCI	AD	All
Age (years)	70.04	72.68	72.47	71.45
Gender (M/F)	6314/11,857	4991/5052	7535/8152	19,702/26,221
Education (years)	16.25	15.73	15.45	15.8
NACCGDS	1.97	3.87	12.56	6.08
CDRSUM	0.11	1.38	6.65	2.66
NACCBMI	82.73	112.77	158.99	115.72
HRATE	118.67	136.29	156.08	135.53
BPSYS	176.68	197.99	208.10	192.32

In light of the large number of features and the sparse data set problem, we selected a subset of features (172 in total) in line with other related studies^33,34^ using the same dataset. Results in selecting a number of features which are informative for the majority of patients, including Subject Demographics, Subject Health History, Physical, Geriatric Depression Scale (GDS), Functional Activities Questionnaire (FAQ), Neuropsychiatric Inventory Questionnaire (NPI-Q), CDR Plus NACC FTLD. Table 8 lists the features used in this study for further investigation. The data size and number of subjects splitting into training and testing sets demonstrated in Supplementary Fig. 7.

#### ADNI

The ADNI dataset was obtained from the ADNI database (http://adni.loni.usc.edu). Established in 2003 as a collaborative effort between the public and private sectors, ADNI’s primary objective is to explore the potential of magnetic resonance imaging, positron emission tomography, biological markers, clinical assessments, and cognitive evaluations for tracking the progression of MCI and early-stage AD. The ADNI dataset serves as an external source for the validation of ML models. The ADNI dataset was pre-processed to align with the NACC dataset, including value mapping and feature name adjustments, as demonstrated in Supplementary Tables 4 and 5. The data size and number of subjects splitting into training and testing sets demonstrated in Supplementary Fig. 8.

**Table 8:** Feature categories and the variable name selected from NACC dataset at the initial stage of the proposed work.

NACC categories	Variable name
Subject demographics	SEX, HISPANIC , HISPOR, HISPORX, RACE, RACEX, RACESEC, RACESECX, RACETER, RACETERX, PRIMLANG, PRIMLANX, EDUC, MARISTAT, NACCLIVS, INDEPEND, RESIDENC, HANDED, NACCAGE, NACCAGEB, NACCNIHR
Physical	WEIGHT, HEIGHT, NACCBMI, BPSYS, BPDIAS, HRATE, VISION, VISCORR, VISWCORR, HEARING, HEARAID, HEARWAID
Subject health history	TOBAC30, TOBAC100, SMOKYRS, PACKSPER, ALCOCCAS, QUITSMOK, ALCFREQ, CVHATT, HATTMULT, HATTYEAR, CVAFIB, CVANGIO, CVBYPASS, CVPACDEF, CVPACE, CVCHF, CVANGINA, CVHVALVE, CVOTHR, CVOTHRX, CBSTROKE, STROKMUL, NACCSTYR, ALCOCCAS, ALCFREQ, HATTMULT, CBTIA, TIAMULT, NACCTIYR, PD, PDYR, PDOTHR, PDOTHRYR, SEIZURES, NACCTBI, TBI, TBIBRIEF, TRAUMBRF, TBIEXTEN, TRAUMEXT, TBIWOLOS, TRAUMCHR, TBIYEAR, NCOTHR, NCOTHRX, DIABETES, DIABTYPE, HYPERTEN, HYPERCHO, B12DEF, THYROID, ARTHRIT, ARTHTYPE, ARTHTYPX, ARTHUPEX, ARTHLOEX, ARTHSPIN, ARTHUNK, INCONTU, INCONTF, APNEA, RBD, INSOMN, OTHSLEEP, OTHSLEEX, ALCOHOL, ABUSOTHR, ABUSX, PTSD, BIPOLAR, SCHIZ, DEP2YRS, DEPOTHR, ANXIETY, OCD, NPSYDEV, PSYCDIS, PSYCDISX’
Geriatric Depression Scale (GDS),	NOGDS, SATIS, DROPACT, EMPTY, BORED, SPIRITS, AFRAID, HAPPY, HELPLESS, STAYHOME, MEMPROB, WONDRFUL, WRTHLESS, ENERGY, HOPELESS, BETTER, NACCGDS
Functional Activities Questionnaire (FAQ),	BILLS, TAXES, SHOPPING, GAMES, STOVE, MEALPREP, EVENTS, PAYATTN, REMDATES, TRAVEL
Neuropsychiatric Inventory Questionnaire (NPI-Q)	NPIQINF, NPIQINFX, DEL, DELSEV, HALL, HALLSEV, AGIT, AGITSEV, DEPD, DEPDSEV, ANX, ANXSEV, ELAT, ELATSEV, APA, APASEV, DISN, DISNSEV, IRR, IRRSEV, MOT, MOTSEV, NITE, NITESEV, APP, APPSEV
CDR®Plus NACC FTLD	MEMORY, ORIENT, JUDGMENT, COMMUN, HOMEHOBB, PERSCARE, COMPORT, CDRLANG
Target Class	NACCUDSD

### Data pre-processing

Given the challenges posed by incomplete data in data analysis and ML model implementation, we have carefully filtered out subjects and attributes with incomplete data. The following steps further describe the data pre-process techniques employed in the current study.

#### Missing values and unmeaningful features

Firstly, variables that exhibit the same value in 90% of the participants are removed, this has reduced number of variables from 172 to 118. Secondly, all variables and subjects (i.e., participants) comprising missing values in more than 50% of their occurrences are removed. This resulted the number of variables to be further reduced to 67. Likewise, the number of records is reduced from 27087 to 26722 for the training set of the CNvsAD subset. We then impute the missing data of the remaining variables using a simple and widely used imputation technique^33^. For continuous variables, mean of the variable was used while for the categorical variables, mode imputation is used. These processes are first applied to the training set then reflected onto the testing set. Supplementary Fig. 1 shows the mean and standard deviation of some feature before and after data imputation to ensure the imputation did not affect the statistics of the features. While Supplementary Table 1 shows the number of participants and imputed values for each data subset.

#### Correlation analysis and data standardisation

Given the nature of data collection within NACC, it is common to encounter a substantial degree of correlation among variables, such as the simultaneous inclusion of RACE and NACCNIHR variables, both of which pertain to a subject’s ethnicity. The inclusion of multiple closely related variables can significantly impact the outcomes reported. As a response, we conducted correlation analysis to identify and eliminate highly correlated features, employing the Cramer’s V correlation method^35^. It’s important to note that Cramer’s V is particularly suitable for categorical features. To address this, we discretized continuous features by categorizing them into bins, drawing inspiration from existing literature. For instance, BMI was categorized into ‘underweight,’ ‘normal,’ ‘overweight,’ and ‘obesity,’ with a similar transformation applied to other continuous features. Detailed conversions of the remaining continuous features are provided in Supplementary Table 2. On the other hand, for categorical features are encoded either using one-hot or label encoding depending on weather the variable is of type nominal (the order of value is not important) or ordinal (order of value is important) respectively.

#### Outlier detection

Outliers are data points that diverge significantly from conventional patterns or are not in accordance with expected normal patterns for the measure under consideration^36^. Despite the importance of this step, several research studies in AD classification ignore this step or may not report it properly. In this study, we utilise two approaches to deal with outliers. Firstly, for categorical features, we calculate the percentage of each value in a variable and then substitute the mode of the variable in all values that have a percentage of less than 3% of the total values. For instance, Supplementary Fig. 2 shows the distribution of values in 9 categorical features, the variable “CDRLANG” has the value of 3 in very few samples of the dataset. Therefore, these values are substituted with value 0 which is the mode of the variables “CDRLANG” as shown in Supplementary Fig. 3. We performed the same operation for the remaining categorical variables.

For the numerical features, we use inter-quartile range (IQR) to identify the outliers within each continuous feature. In IQR, the interest falls on the lower quartile (Q1) and the upper quartile (Q3), where IQR is calculated as follows:

1 $$\begin{aligned} IQR = Q3-Q1 \end{aligned}$$

Outliers are then identified using Eqs. (2) and (3), representing a decision threshold where the data points falling outside the range are treated as outliers. The decision range is calculated as follows:

2 $$\begin{aligned} Lower\, bond = (Q1 -1.5 * IQR) \end{aligned}$$

3 $$\begin{aligned} Upper\, bond = (Q1 +1.5 * IQR) \end{aligned}$$

The term outlier in this study refers to data points that fall outside the Lower Bound or that exceed the Upper Bound. Supplementary Fig. 4 shows a boxplot for the continuous variables in their original form. It can be noticed that the distribution of data points improved after IQR-based outlier removal as shown in Supplementary Fig. 5.

### Feature selection and dimensionality reduction

Although several features are filtered out during the initial preprocessing step, the number of remaining features is still substantial. Generally, the ML models trained over reduced but relevant set of features, result in both reduction in computation costs and, in most cases, performance improvement^37^. In this study, we employed the Boruta algorithm^38^ for the feature selection which is based on the RF algorithm and determines feature importance by comparing the original features with shuffled and permuted versions. The algorithm iteratively selects and rejects features based on their importance scores until a stable set of relevant features is obtained and is particularly useful in high-dimensional datasets (as in our case) where feature selection is crucial for model explainability and performance. Algorithm 1 presents the steps we use to to eliminate the less significant features.

**Algorithm 1:**
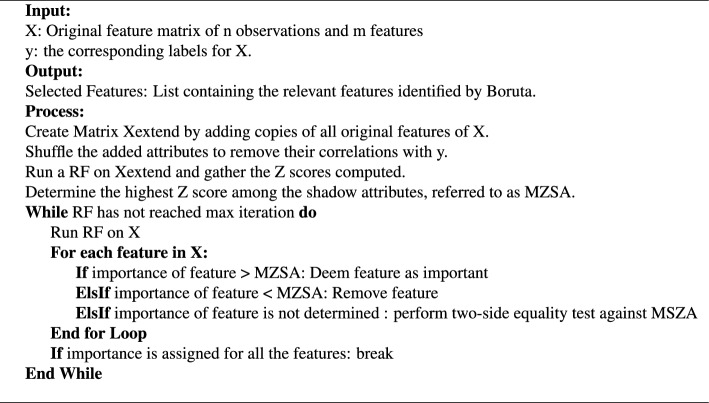
Boruta algorithm.

Table 9 shows the selected features for each data subset identified as most relevant using the Algorithm 1 . It can be noticed that the number of identified features are substantially reduced to 24 only (compared to 64 in original NC vs AD data subset). Furthermore, seven of the selected features belong to CDR which measures the relative severity of dementia by assigning a score between 0 (no impairment) and 3 (severe impairment)^39^. A clinician’s clinical judgment and a semi structured interview with the subject and caregiver (informant) determine CDR score. On the other hand, nine features fall with FAQ which measures difficulty with daily living activities and was found to be a valid and reliable measure according to studies in the literature^40^. Five features among the selected ones belong to the NPI-Q which was developed by Cummings^41^, to assess behavioural symptoms associated with dementia and found to be an effective tool for the assessment of dementia in different populations^42,43^. Two feature belongs to GDS and one from subject’s Demographics.

In contrast to NC vs AD data subset, Algorithm 1 identified only 10 features as important for NC vs MCI subset. Out of these, six aligns with the CDR, two with GDS and two with FAQ. Similarly, for the MCI vs AD subset, 17 features are identified as informative. Finally, 18 variables were selected for multi-class category NC vs MCI vs AD. The selected variables for each data subset are shown in Table 9. Across all classification tasks, we consistently observe a shared set of features, namely MEMORY, ORIENT, JUDGMENT, COMMUN, CDRLANG, MEMPROB, NACCGDS, BILLS, and TAXES. These features consistently demonstrate their significance in distinguishing cognitive states, emphasizing their crucial role in AD diagnosis. In addition to these common features, the NC vs. AD classification task incorporates task-specific features such as COMPORT, AGIT, ANX, APA, IRR, and MOT. Notably, the inclusion of features related to behavioural domains (AGIT, ANX, APA, IRR, and MOT) gains importance when classifying NC vs. AD. Additionally, it is noteworthy that the feature HOMEHOBB is shared among all tasks, except in the case of NC vs. AD. This distinctive pattern further emphasizes the importance of certain features in differentiating between cognitive states.

To externally validate the ML classifiers, we incorporated data from ADNI. However, it’s noteworthy that three features, namely COMPORT, CDRLANG, and INDEPEND, were not present in the ADNI dataset (refer to Supplementary Figs. 9, 10, 11, and 12 for final feature sets). Consequently, we opted to exclude these features. Subsequently, we trained the ML classifiers on the remaining selected features and proceeded with the external evaluation of the classifiers using the ADNI dataset.

**Table 9:** Informative features selected by the Algorithm 1 for each data subset.

Data subset	Selected features
NC vs AD	MEMORY, ORIENT, JUDGMENT, COMMUN, PERSCARE, COMPORT, CDRLANG, MEMPROB, NACCGDS, AGIT, ANX, APA, IRR, MOT, BILLS, TAXES, SHOPPING, GAMES, STOVE, MEALPREP, EVENTS,PAYATTN, TRAVEL, INDEPEND
NC vs MCI	MEMORY, ORIENT, JUDGMENT, COMMUN, HOMEHOBB, CDRLANG, MEMPROB, NACCGDS, BILLS, TAXES
MCI vs AD	MEMORY, ORIENT, JUDGMENT, COMMUN, HOMEHOBB, PERSCARE, CDRLANG, NACCGDS, BILLS, TAXES, SHOPPING, GAMES, MEALPREP, EVENTS, PAYATTN, TRAVEL, INDEPEND
NC vs MCI vs AD	MEMORY, ORIENT, JUDGMENT, COMMUN, HOMEHOBB, CDRLANG, MEMPROB, NACCGDS, BILLS, TAXES, SHOPPING, GAMES, STOVE, MEALPREP, EVENTS, PAYATTN, TRAVEL, INDEPEND

### Model explanation

The ML algorithms such as tree-based ensembles or neural networks are well-known for their powerful predictive performance however, highly complex prediction mechanisms are associated with such methods. Because of the extensive computations, these models are considered as black boxes, limiting their usefulness particularly in fields like healthcare where the explainability of decisions holds significant importance. In order to serve for the model’s explainability, we utilise methods for the rule extraction to unlock the hidden knowledge of a ML model towards final decision making.

#### Stable and interpretable rule set for classification—random forest based rule classification

In this study, we use SIRUS^44^, SIRUS is specifically designed as a stable predictive algorithm. It utilizes a modified version of RF to generate a substantial number of rules. Among these rules, SIRUS selects those that surpass a certain redundancy threshold represented by the tuning hyperparameter p0. using cross-validation to determine the best hyperparameter, which determines the number of relevant rules to extract. The optimal value of this hyperparameter was estimated by considering the proportion of times a rule appeared among the trees of the RF model. Only rules that met this criterion were considered relevant and included in the extraction process.The algorithm and technical formulation of SIRUS can be found in the original work^44^.SIRUS was used in various research and found to be an effective and stable model^45–48^.

#### Class rule mining

As a special case of conventional rule mining^49^, we use CARs, with target classes used as a consequence. CARs are commonly used to identify frequent patterns in large datasets that can be readily interpreted by humans. In most cases, confidence (c) and support (s) metrics are used to determine the strength of a rule (X) and therefore the strength of its association where support is mathematically define in Eq. (4):

4 $$\begin{aligned} s(X \Rightarrow Y) = \frac{frq(X \cup Y )}{N} \end{aligned}$$

Where N indicates how many observations/records there are in the dataset. In a rule, confidence (c) represents the probability that factor Y occurs when factor X is present and defined mathematically in Eq. (5).

5 $$\begin{aligned} c(X \Rightarrow Y) = \frac{frq(X \cup Y )}{frq(X)} \end{aligned}$$

Rules are typically evaluated by varying thresholds for ‘c’ and ‘s’ criteria^50^. These metrics however, can misinterpret the significance or importance of an association because only the popularity of X is considered, not that of Y. An additional measure called lift accounts for the popularity of each constituent item (i.e., X and Y), which indicates how the X affects the Y, and is calculated as follows (Eq. 6):

6 $$\begin{aligned} lift(X \Rightarrow Y) = \frac{s(X \cup Y )}{s(X)*s(Y)} \end{aligned}$$

Here, X and Y are independent when $lift(XY)=1$, whereas $lift(XY)>1$ indicates that they are positively dependent. A detailed explanation of CARs can be found in study^51^. Rule mining methods are employed in various work^52–54^.

### Experimental design

The current work utilises a composite of algorithms to identify the most significant features from NACC dataset as well as, extract human understandable rules for AD classification to serve for the explainability of ML models. In each of the following experiments, the dataset is divided into 80% of samples for training while the rest of the samples are reserved to evaluate the model (i.e., unseen data).

Experiment 1 (EXP1) is essential for determining the efficacy of ML models in classifying clinical stages of cognitive impairment using the NACC dataset. To ensure accurate results, pre-processing steps must be conducted first followed by the training and evaluation of ML models as shown in Fig. 3. This experiment is conducted to assess the ability of ML models to accurately classify the clinical stages with 64, 55, 67, 66 features (i.e. before feature selection) for NC vs AD, NC vs MCI, MCI vs AD and NC vs MCI vs AD, respectively.

In Experiment 2 (EXP2), ML models with same configuration as EXP1 are trained and evaluated over the reduced feature set (i.e., using the only important features only as identified by Algorithm 1 resulting in selecting 24 feature for NC vs AD subset, 10 for NC vs MCI subset, 17 for MCI vs AD subset and 18 for NC vs MCI vs AD subset (Table 9). This experiment is performed to determine the effectiveness of our feature selection algorithm for the purpose of classifying AD.

Experiment 3 (EXP3) is a continuation of EXP2, in which the same features are used to train and test the ML models for each data subset. However, instead of classifying the cognitive state of an individual at the baseline visit, the task of the ML models is to predict the cognitive state of an individual after four years of baseline visit. This is a crucial experiment, as it will help to determine whether the features that have been identified as important (in Table 9), are effective in predicting the cognitive state of an individual over a longer period of time.

Experiment 4 (EXP4), we aimed to evaluate the generalization capabilities of the top-performing models developed in EXP2 and EXP3. This evaluation was conducted using an external dataset, specifically the ADNI dataset. Our objective was to assess the classifiers’ ability to generalize across different datasets for both classification and prediction tasks.

Experiment 5 (EXP5), we utilised the CAR and SIURS algorithms to extract the human understandable rules capture significant patterns within the data. The results of this experiment will hold a substantial value as they provide insights into the cognitive state of an individual. To ensure the robustness of the selected features, the results obtained from CAR and SIURS are compared to those of the SHAP and LIME models, further enhancing the validation of the findings

### Supplementary Information

Supplementary Information.

## Data Availability

The NACC and ADNI Datasets are openly available for researchers at https://naccdata.org/ and http://adni.loni.usc.edu with a data access request.

## Code availibility

The source code used to generate the results in this article is publicly available at: https://github.com/AbbasAlatrany/ML_AD_research.
