# Supplementary material for: An explainable machine learning approach for Alzheimer’s disease classification
Source: Sci Rep. 2024 Feb 1;14:2637. doi: 10.1038/s41598-024-51985-w (PMC10834965; doi:10.1038/s41598-024-51985-w)
Supplement: Supplementary file 1 — Supplementary Information. [file 41598_2024_51985_MOESM1_ESM.pdf]

**An Explainable Machine Learning Approach for Alzheimer's Disease Classification**

**Abbas Saad Alatrany, Wasiq Khan, Abir Hussain, Hoshang Kolivand and Dhiya Al-Jumeily**

**Supplementary Materials**

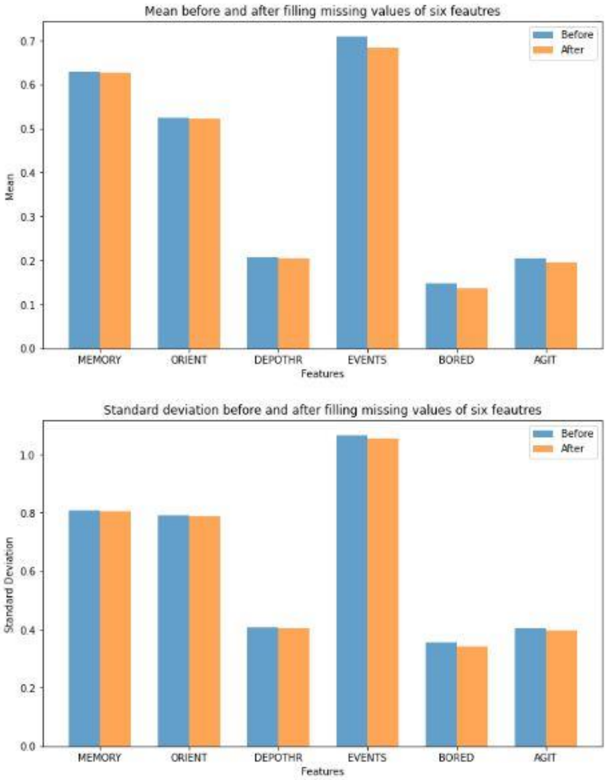


**Supplementary Figure 1**: Mean and Standard deviation for some variables of NC vs AD training subset before and after filling missing values.

**Supplementary Table 1**: Numbers of participants with imputed data and imputed values for each dataset.

| Data Subset | Number of Participants | Participants with imputed Data | No. of Non-Missing Values | No. of Imputed Values |
|---|---|---|---|---|
| NC vs AD Training set | 27,087 | 18,667 | 1,851,437 | 90,115 |
| NC vs AD Testing set | 6,771 | 4,522 | 469,606 | 20,635 |
| NC vs MCI Training set | 22,572 | 13,621 | 1,348,079 | 40,875 |
| NC vs MCI Testing set | 5,642 | 9,249 | 343,093 | 9,249 |

| | | | | |
|---|---|---|---|---|
| MCI vs AD Training set | 20,584 | 15,793 | 1,494,011 | 83,520 |
| MCI vs AD Testing set | 5,146 | 3,903 | 375,781 | 19,192 |
| NC vs MCI vs AD Training set | 35,121 | 24,351 | 24,11,249 | 112,823 |
| NC vs MCI vs AD Testing set | 8,780 | 5,970 | 606,386 | 26,309 |

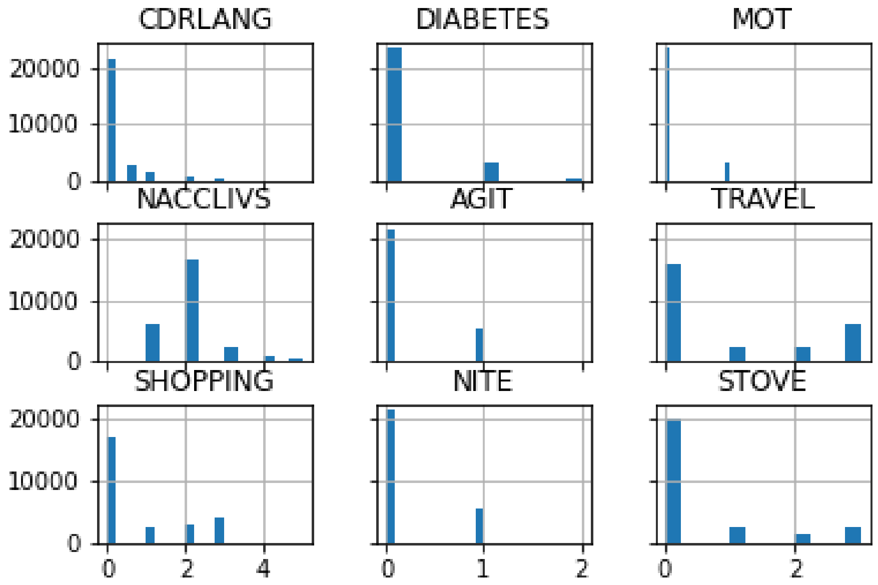


**Supplementary Figure 2:** The distribution of values of some variables of NC vs AD training dataset.

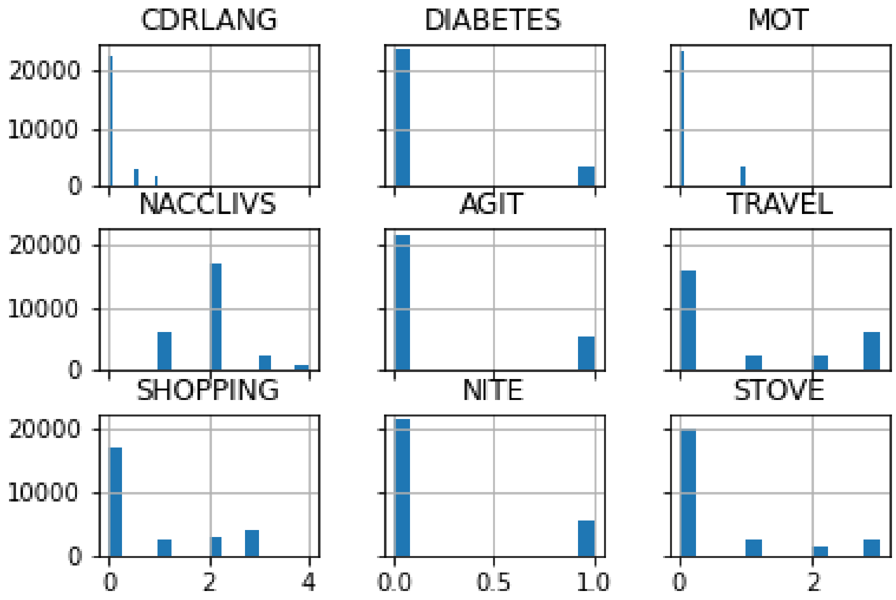


**Supplementary Figure 3:** The distribution of values of some categorical features from NC vs AD training subset after substituting the mode of the feature instead of the values that account of 3% of the feature.

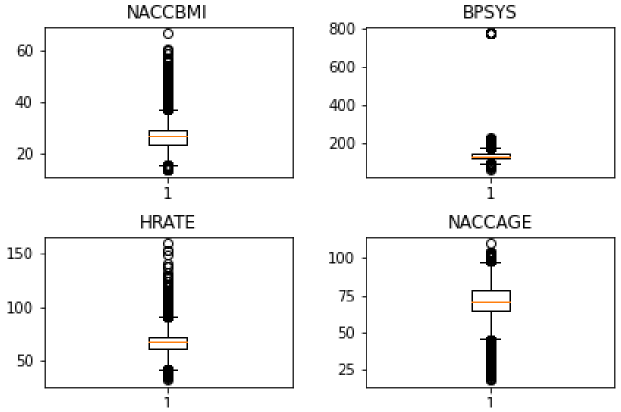


**Supplementary Figure 4:** *Boxplot to show the distribution of data points of continuous variables from NC vs AD training subset.*

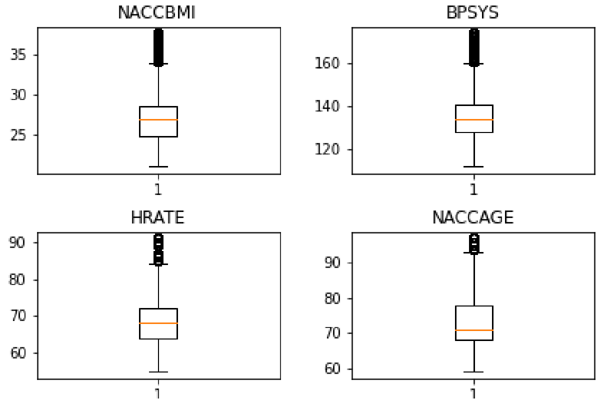


**Supplementary Figure 5:** Boxplot to show the distribution of data points of continuous variables after removing outlier data points from NC vs AD training subset.

**Supplementary Table 2:** discretized continuous values inspired by literature. * Years of education converted into no Bachelor's degree (0), with Bachelor's degree (1), with a postgraduate degree. **Years of smoking converted into bins depending on quantile analysis.

| Feature Name | Categories bins |
| --- | --- |
| NACCAGE[1] | >60, 60 - 75, >75], labels=[0,1,2] |
| NACCBMI[2] | <18.5, 18.5 - 25, 25 - 30, >30], labels=[0,1, 2,3] |
| BPSYS[3] | <90, 90 - 140, >140, labels=[0,1, 2] |
| BPDIAS[3] | <60, 60 - 90, > 90, labels=[0,1, 2] |
| EDU* | <12, 12 – 16 , > 16, labels=[0,1, 2] |
| HRATE [4] | <60, 60 - 100, >100, labels=[0,1,0] |
| SMOKYRS** | <15, 15 - 30, >30, labels=[0,1, 2] |

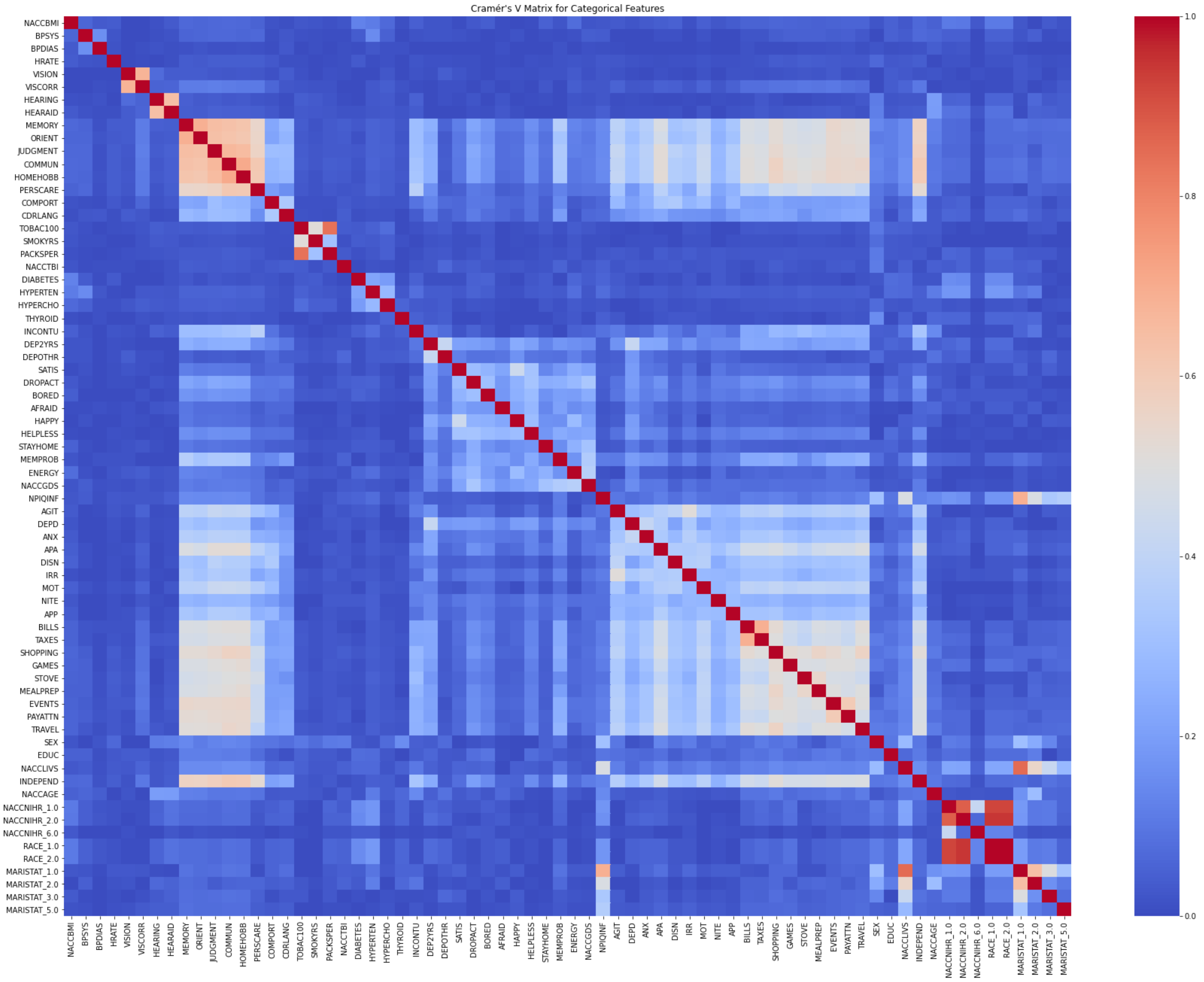


**Supplementary Figure 6**: Correlation matrix of CN vs AD training subset.

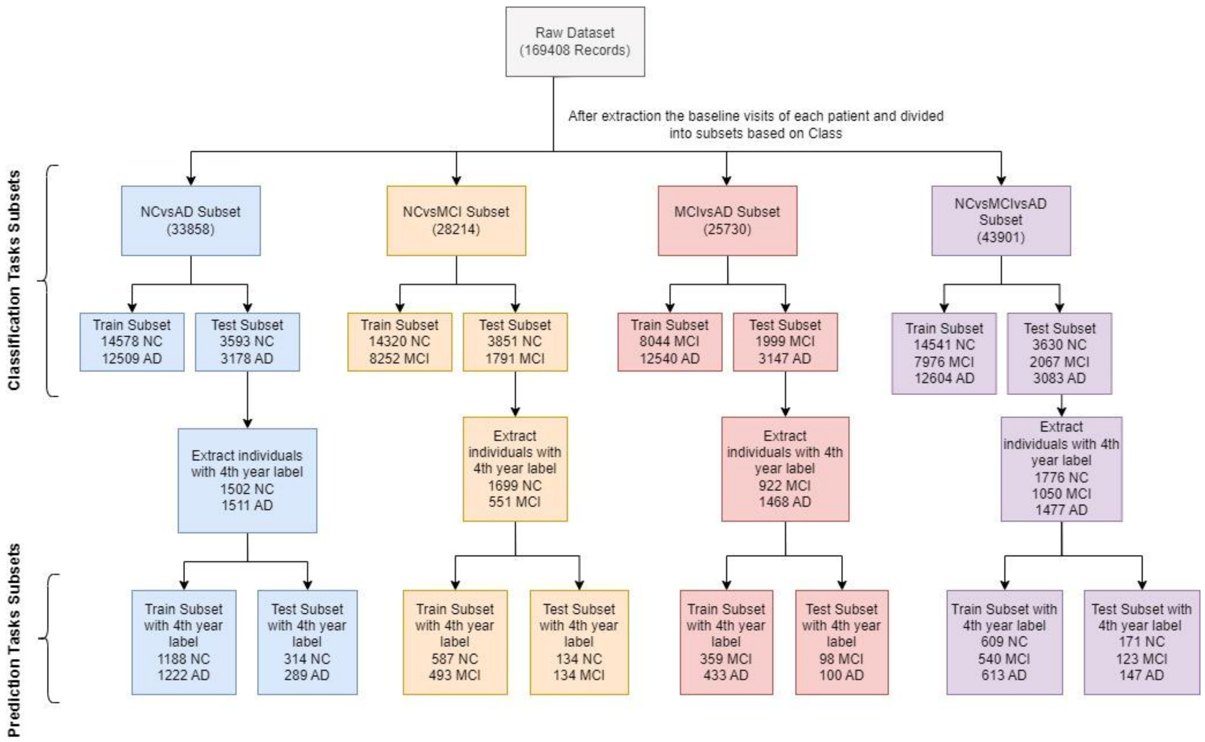


**Supplementary Figure 7**: The sizes of the NACC data subsets for each task. In the prediction tasks concerning NC vs MCI, MCI vs AD, and NC vs MCI vs AD, a downsizing approach was applied to randomly select samples from the NC and AD classes. This selection process aimed to match the size of the MCI class, addressing the issue of class imbalance.

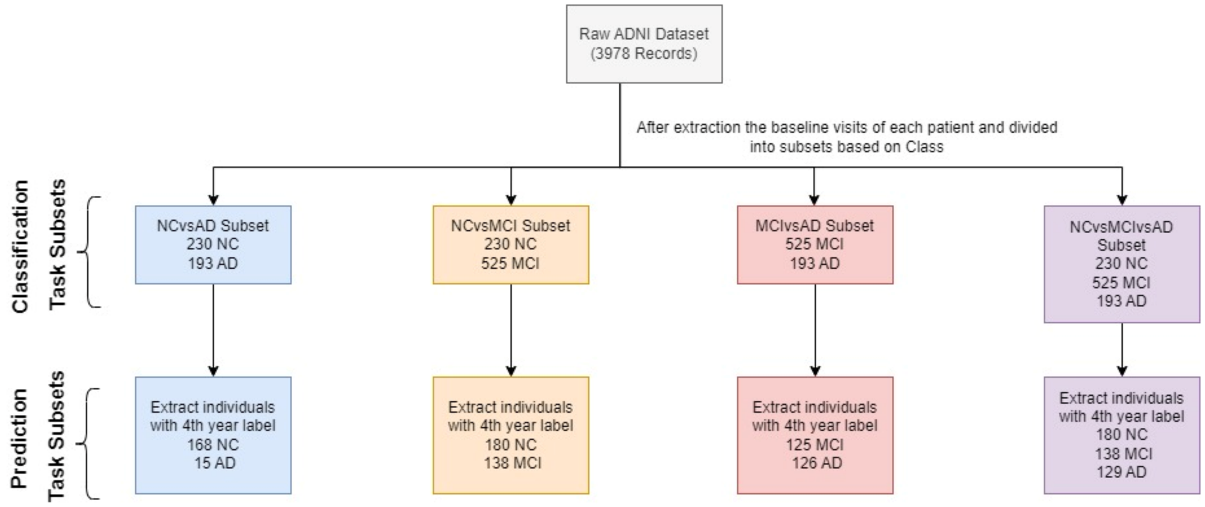


**Supplementary Figure 8:** The sizes of the ADNI data subsets used for external validation of the trained models. The class imbalance in this subset did not affect the model, as the model was trained on balanced data from the NACC dataset.

a)

'NACCBMI', 'BPSYS', 'BPDIAS', 'HRATE', 'VISION', 'VISCORR', 'HEARING', 'HEARAID', 'MEMORY', 'ORIENT', 'JUDGMENT', 'COMMUN', 'PERSCARE', 'COMPORT', 'CDRLANG', 'TOBAC100', 'SMOKYRS', 'NACCTBI', 'DIABETES', 'HYPERTEN', 'HYPERCHO', 'THYROID', 'INCONTU', 'DEP2YRS', 'DEPOTHR', 'SATIS', 'DROPACT', 'BORED', 'AFRAID', 'HAPPY', 'HELPLESS', 'STAYHOME', 'MEMPROB', 'ENERGY', 'NACCGDS', 'NPIQINF', 'AGIT', 'DEPD', 'ANX', 'APA', 'DISN', 'IRR', 'MOT', 'NITE', 'APP', 'BILLS', 'TAXES', 'SHOPPING', 'GAMES', 'STOVE', 'MEALPREP', 'EVENTS', 'PAYATTN', 'TRAVEL', 'SEX', 'EDUC', 'NACCLIVS', 'INDEPEND', 'NACCAGE', 'NACCNIHR_1.0', 'NACCNIHR_6.0', 'MARISTAT_2.0', 'MARISTAT_3.0', 'MARISTAT_5.0'

↓ Features Selected from CNvsAD training data subset using Brouta algorithm.

b)

'MEMORY', 'ORIENT', 'JUDGMENT', 'COMMUN', 'PERSCARE', 'COMPORT', 'CDRLANG', 'MEMPROB', 'NACCGDS', 'AGIT', 'ANX', 'APA', 'IRR', 'MOT', 'BILLS', 'TAXES', 'SHOPPING', 'GAMES', 'STOVE', 'MEALPREP', 'EVENTS', 'PAYATTN', 'TRAVEL', 'INDEPEND'

↓ Three features removed due to not available in ADNI dataset.

c)

'MEMORY', 'ORIENT', 'JUDGMENT', 'COMMUN', 'PERSCARE', 'MEMPROB', 'NACCGDS', 'AGIT', 'ANX', 'APA', 'IRR', 'MOT', 'BILLS', 'TAXES', 'SHOPPING', 'GAMES', 'STOVE', 'MEALPREP', 'EVENTS', 'PAYATTN', 'TRAVEL'

**Supplementary Figure 9:** Features for CN vs AD subset: a) after data pre-processing, b) after feature selection, c) final selected features after remove feature which are not available in ADNI dataset.

a)

'NACCBMI', 'BPSYS', 'BPDIAS', 'HRATE', 'VISION', 'VISCORR', 'HEARING','HEARAID', 'MEMORY', 'ORIENT', 'JUDGMENT', 'COMMUN', 'HOMEHOBB','CDRLANG', 'TOBAC100', 'SMOKYRS', 'CVOTHR', 'NACCTBI', 'DIABETES','HYPERTEN', 'HYPERCHO', 'THYROID', 'INCONTU', 'DEP2YRS', 'DEPOTHR', 'SATIS', 'DROPACT', 'BORED', 'AFRAID', 'HAPPY', 'STAYHOME', 'MEMPROB','ENERGY', 'NACCGDS', 'NPIQINF', 'AGIT', 'DEPD', 'ANX', 'APA', 'IRR','NITE', 'BILLS', 'TAXES', 'TRAVEL', 'SEX', 'EDUC', 'NACCLIVS', 'HANDED','NACCAGE', 'NACCNIHR_1.0', 'NACCNIHR_6.0', 'RACE_5.0', 'MARISTAT_2.0', 'MARISTAT_3.0'. 'MARISTAT_5.0'

↓ Features Selected from CNvsMCI training data subset using Brouta algorithm.

b)

'MEMORY', 'ORIENT', 'JUDGMENT', 'COMMUN', 'HOMEHOBB', 'CDRLANG', 'MEMPROB', 'NACCGDS', 'BILLS', 'TAXES’

↓ One feature removed due to not available in ADNI dataset.

c)

'MEMORY', 'ORIENT', 'JUDGMENT', 'COMMUN', 'HOMEHOBB', 'MEMPROB', 'NACCGDS', 'BILLS', 'TAXES’

**Supplementary Figure 10**: Features for CN vs MCI subset: a) after data pre-processing, b) after feature selection, c) final selected features after remove feature which are not available in ADNI dataset.

a)

'NACCBMI', 'BPSYS', 'BPDIAS', 'HRATE', 'VISION', 'HEARING', 'HEARAID','MEMORY', 'ORIENT', ‘JUDGMENT', 'COMMUN', 'HOMEHOBB', 'PERSCARE', 'COMPORT', 'CDRLANG', 'TOBAC100', 'SMOKYRS', 'NACCTBI', 'DIABETES', 'HYPERTEN', 'HYPERCHO', 'THYROID', 'INCONTU', 'DEP2YRS', 'DEPOTHR', 'SATIS', 'DROPACT', 'EMPTY', 'BORED', 'AFRAID', 'HAPPY', 'HELPLESS', 'STAYHOME', 'MEMPROB', 'WRTHLESS', 'ENERGY', 'BETTER', 'NACCGDS','NPIQINF', 'DEL', 'AGIT', 'DEPD', 'ANX', 'APA', 'DISN', 'IRR', 'MOT', 'NITE', 'APP', 'BILLS', 'TAXES', 'SHOPPING', 'GAMES', 'STOVE', 'MEALPREP', 'EVENTS', 'PAYATTN', 'TRAVEL'. 'SEX'. 'EDUC'. 'NACCLIVS'.

Features Selected from MCIvsAD training data subset using Brouta algorithm.

b)

'MEMORY', 'ORIENT', 'JUDGMENT', 'COMMUN', 'HOMEHOBB', 'PERSCARE', 'CDRLANG', 'NACCGDS', 'BILLS', 'TAXES', 'SHOPPING', 'GAMES', 'MEALPREP', 'EVENTS', 'PAYATTN', 'TRAVEL', 'INDEPEND'

Two features removed due to not available in ADNI dataset.

c)

'MEMORY', 'ORIENT', 'JUDGMENT', 'COMMUN', 'HOMEHOBB', 'PERSCARE', 'NACCGDS', 'BILLS', 'TAXES', 'SHOPPING', 'GAMES', 'MEALPREP', 'EVENTS', 'PAYATTN', 'TRAVEL'

**Supplementary Figure 11:** Features for MCI vs AD subset: a) after data pre-processing, b) after feature selection, c) final selected features after remove feature which are not available in ADNI dataset.

a)

'NACCBMI', 'BPSYS', 'BPDIAS', 'HRATE', 'VISION', 'VISCORR', 'HEARING','HEARAID', 'MEMORY', 'ORIENT', 'JUDGMENT', 'COMMUN', 'HOMEHOBB','PERSCARE', 'COMPORT', 'CDRLANG', 'TOBAC100', 'SMOKYRS', 'NACCTBI','DIABETES', 'HYPERTEN', 'HYPERCHO', 'THYROID', 'INCONTU', 'DEP2YRS', 'DEPOTHR', 'SATIS', 'DROPACT', 'BORED', 'AFRAID', 'HAPPY', 'HELPLESS','STAYHOME', 'MEMPROB', 'ENERGY', 'NACCGDS', 'NPIQINF', 'AGIT', 'DEPD','ANX', 'APA', 'DISN', 'IRR', 'MOT', 'NITE', 'APP', 'BILLS', 'TAXES','SHOPPING', 'GAMES', 'STOVE', 'MEALPREP', 'EVENTS', 'PAYATTN', 'TRAVEL','SEX', 'EDUC', 'NACCLIVS'. 'INDEPEND'. 'HANDED'. 'NACCAGE'.

Features Selected from NCvsMCIvsAD training data subset using Brouta algorithm.

b)

'MEMORY', 'ORIENT', 'JUDGMENT', 'COMMUN', 'HOMEHOBB', 'CDRLANG','MEMPROB', 'NACCGDS', 'BILLS', 'TAXES', 'SHOPPING', 'GAMES', 'STOVE','MEALPREP', 'EVENTS', 'PAYATTN', 'TRAVEL', 'INDEPEND'

Two features removed due to not available in ADNI dataset.

c)

'MEMORY', 'ORIENT', 'JUDGMENT', 'COMMUN', 'HOMEHOBB','MEMPROB', 'NACCGDS', 'BILLS', 'TAXES', 'SHOPPING', 'GAMES', 'STOVE','MEALPREP', 'EVENTS', 'PAYATTN', 'TRAVEL'

**Supplementary Figure 12:** Features for CN vs MCI vs AD subset: a) after data pre-processing, b) after feature selection, c) final selected features after remove feature which are not available in ADNI dataset.

**Supplementary Table 3:** performance of classifiers for the classification of NC vs AD using subject demographics and patient history features only.

| ML Model | Accuracy% | Precision% | Recall% | F1 score% |
|---|---|---|---|---|
| RF | 87.7 | 91.5 | 80.4 | 85.6 |
| KNN | 87.1 | 91.9 | 78.6 | 84.8 |
| NB | 86.2 | 88.6 | 79.9 | 84 |
| SVM | 88.7 | 96.8 | 77.9 | 86.3 |

**Supplementary Table 4**: Mapping values of ADNI dataset features to match corresponding values of matching feature of NACC dataset using the ADNI and NACC datasets dictionaries. Since the downloaded data from ADNI has text as values in the features step inmoved mapping these text to corresponding integers.

| Feature Names | Mapping values |
|---|---|
| 'FAQFINAN','FAQFORM','FAQSHOP',<br>'FAQGAME','FAQBEVG','FAQMEAL',<br>'FAQEVENT','FAQTV','FAQTRAVL' | 'Normal (0)': 0,<br>'Never did, but could do now (0)':0,<br>'Never did, would have difficulty now (1)': 1,<br>'Has difficulty, but does by self (1)': 1,<br>'Requires assistance (2)': 2,<br>'Dependent (3)': 3} |
| 'GDMEMORY' | 'No(0)': 0,<br>'Yes(1)': 1 |
| 'NPIC','NPIE','NPIG','NPII','NPIJ' | 'No': 0,<br>'Yes': 1 |
| 'DX'- Class label | 'CN': 0,<br>'MCI': 1,<br>'Dementia':2 |

**Supplementary Table 5**: Conversion of ADNI feature names to match the corresponding feature names in the NACC dataset to ensure compatibility with ML classifiers.

| ANDI Feature Name | NACC feature Name |
|---|---|
| 'CDMEMORY' | 'MEMORY' |
| 'CDORIENT' | 'ORIENT' |
| 'CDJUDGE' | 'JUDGMENT' |
| 'CDCOMMUN' | 'COMMUN' |
| 'CDHOME' | 'HOMEHOBB' |
| 'CDCARE' | 'PERSCARE' |
| 'GDMEMORY' | 'MEMPROB' |
| 'GDTOTAL' | 'NACCGDS' |
| 'NPIC' | 'AGIT' |
| 'NPIE' | 'ANX' |
| 'NPIG' | 'APA' |
| 'NPII' | 'IRR' |

| | |
|---|---|
| 'NPIJ' | 'MOT' |
| 'FAQFINAN' | 'BILLS' |
| 'FAQFORM' | 'TAXES' |
| 'FAQSHOP' | 'SHOPPING' |
| 'FAQGAME' | 'GAMES' |
| 'FAQBEVG' | 'STOVE' |
| 'FAQMEAL' | 'MEALPREP' |
| 'FAQEVENT' | 'EVENTS' |
| 'FAQTV' | 'PAYATTN' |
| 'FAQTRAVL' | 'TRAVEL' |


1 Lachman, M. E. in *International Encyclopedia of the Social & Behavioral Sciences* (eds Neil J. Smelser & Paul B. Baltes) 135-139 (Pergamon, 2001).

2 Juraschek, S. P., Miller III, E. R. & Gelber, A. C. Body mass index, obesity, and prevalent gout in the United States in 1988–1994 and 2007–2010. *Arthritis care & research* **65**, 127-132 (2013).

3 Jones, N. R., McCormack, T., Constanti, M. & McManus, R. J. Diagnosis and management of hypertension in adults: NICE guideline update 2019. *British Journal of General Practice* **70**, 90-91 (2020).

4 Avram, R. *et al.* Real-world heart rate norms in the Health eHeart study. *NPJ Digit Med* **2**, 58, doi:10.1038/s41746-019-0134-9 (2019).
